# IgG antibody response against *Anopheles* salivary gland proteins in asymptomatic *Plasmodium* infections in Narino, Colombia

**DOI:** 10.1186/s12936-020-3128-9

**Published:** 2020-01-23

**Authors:** Jehidys Montiel, Luisa F. Carbal, Alberto Tobón-Castaño, Gissella M. Vásquez, Michael L. Fisher, Berlin Londono-Rentería

**Affiliations:** 1 Grupo Malaria, Universidad de Antioquia, Medellín, Colombia; 2 Department of Entomology, Kansas State University, Manhattan, KS USA; 3 Facultad de Medicina, Instituto de Investigaciones Medicas, Universidad de Antioquia, Medellín, Colombia; 4 U.S. Naval Medical Research Unit No. 6 (NAMRU-6), Lima, Peru

**Keywords:** Asymptomatic malaria, *An.* (*Nys.*) *albimanus*, *An.* (*Nys.*) *darlingi*, Antibodies, Bite exposure

## Abstract

**Background:**

The humoral immune response against *Anopheles* salivary glands proteins in the vertebrate host can reflect the intensity of exposure to *Anopheles* bites and the risk of *Plasmodium* infection. In Colombia, the identification of exposure biomarkers is necessary due to the several *Anopheles* species circulating. The purpose of this study was to evaluate risk of malaria infection by measuring antibody responses against salivary glands extracts from *Anopheles* (*Nyssorhynchus*) *albimanus* and *Anopheles* (*Nys.*) *darlingi* and also against the gSG6-P1 peptide of *Anopheles gambiae* in people residing in a malaria endemic area in the Colombian Pacific coast.

**Methods:**

Dried blood spots samples were eluted to measure the IgG antibodies against salivary gland extracts of *An. albimanus* strains STECLA (STE) and Cartagena (CTG) and *An. darlingi* and the gSG6-P1 peptide by ELISA in uninfected people and microscopic and submicroscopic *Plasmodium* carriers from the Colombia Pacific Coast. A multiple linear mixed regression model, Spearman correlation, and Mann–Whitney U-test were used to analyse IgG data.

**Results:**

Significant differences in specific IgG levels were detected between infected and uninfected groups for salivary glands extracts from *An. albimanus* and for gSG6-P1, also IgG response to CTG and gSG6-P1 peptide were positively associated with the IgG response to *Plasmodium falciparum* in the mixed model.

**Conclusion:**

The CTG and STE *An. albimanus* salivary glands extracts are a potential source of new *Anopheles* salivary biomarkers to identify exposure to the main malaria vector and to calculate risk of disease in the Colombian Pacific coast. Also, the gSG6-P1 peptide has the potential to quantify human exposure to the subgenus *Anopheles* vectors in the same area.

## Background

Malaria is caused by the protozoan parasite *Plasmodium* and is transmitted by female *Anopheles* mosquitoes. Although significant advances have been made towards its elimination in several previously endemic countries, malaria remains a significant public health concern [1]. The World Malaria Report in 2018 estimated that the global burden of malaria comprised around 219 million reported cases and 435,000 deaths worldwide [2]. Specifically, in Colombia, there was a decrease in the estimated number of malaria cases by more than 20% between 2016 and 2017 [2]. Despite this, malaria remains one of the foremost public health concerns in some states in Colombia such as Nariño, which is located along the Pacific coast of the country. In 2017, 26% of malaria cases in Colombia came from Nariño where, unlike other regions, *Plasmodium falciparum* is the most common species (96.3%) [3].

More than 47 *Anopheles* species in five subgenera have been reported in Colombia [4]. The majority of primary malaria vectors in Colombia belong to the subgenus *Nyssorhynchus*, with *Anopheles* (*Nys.*) *nuneztovari, Anopheles* (*Nys.*) *albimanus* and *Anopheles* (*Nys.*) *darlingi* as the most important malaria vectors in areas of high malaria transmission [5]. On the South Pacific coast, several species has been associated with malaria transmission with *An. albimanus* is the main vector [6, 7]. Previous studies reported that the *An. albimanus* lineage circulating the Southern region may be different from the one found the in the Northern part of the country suggesting that two different lineages are circulating in the country [7–9]. Interestingly, malaria prevalence in these sites is significantly different and further studies evaluating vector competence and susceptibility to both, *Plasmodium vivax* and *P. falciparum* [7] as well as to measure potential changes in salivary content that could impact pathogen transmission [10] are necessary.

Extensive entomological research has been done in the Nariño Department [7, 11, 12]. This research suggests that mosquitoes from the subgenus *Anopheles*, *Anopheles* (*An.*) *calderoni* and *Anopheles* (*An.*) *punctimacula* are also important malaria vectors in the area. However, these two species are often misclassified due to their high morphological similarities [11]. However, *An. calderoni* was found infected with both *P. vivax* and *P. falciparum* with an annual entomological inoculation rate (EIR) of 2.84 bites/human/year in Nariño between 2012 and 2013 [11]. Also, a previous study reported EIR for *An. calderoni* between 1.7 and 14.7 from 2009 to 2010, while EIR reported for *An. albimanus* during the same period was found between 0.1 and 2.6 [12]. Suggesting that *An. calderoni* is a primary vector of malaria in Nariño. Furthermore, in the Tumaco city, located in the Narino Department), Ahumada et al., reported different malaria incidence in places where *An. albimanus* and *An. calderoni* were found in the 2011–2012 study. Specifically, they reported a high Annual Parasite Index (API) (73 cases/1000 inhabitant) in places where *An. calderoi* is the predominant species compared to lower (27 cases/1000) where *An. albimanus* was predominant [7].

To design a proper vector control method, it is necessary to accurately determine human-vector interaction and the proportion of those vectors that are infected. Vectorial capacity (VC) and EIR are quantitative entomological indicators used to determine epidemiology of vector-borne diseases such as malaria. The VC is used as the measure of a mosquito population’s proficiency to transmit an infectious agent to a susceptible population [13], while EIRs are useful to establish a direct estimation of transmission risk [14, 15]. In the case of malaria, the EIR is the gold standard for measuring transmission intensity. EIRs are based on the number of mosquitoes captured and the proportion of mosquitoes infected with *Plasmodium* [16]. However, estimation of EIR is expensive and may be insufficient in areas of low or seasonal transmission [17, 18]. Human Landing Collection (HLC) is currently the only mosquito catching method that can directly measure the biting rates of human-seeking mosquitoes. Unfortunately, it is only applicable to mosquitoes seeking human adults and results are difficult to extrapolate to children or to pregnant women that are the most vulnerable to malaria [19]. Furthermore, during HLC, the human bait is exposed to the diseases transmitted by the landing mosquitoes posing ethical concerns on implementation of this technique [20]. As an alternative, catching traps such as the CDC (Center for Disease Control) light trap and the bed net traps have been developed and the data collected is useful in estimating vector populations when the studies are properly controlled. However, these trapping methods often differ in the number of host-seeking mosquito population sampled [21]. Still, in spite the high number of mosquitoes captured on these studies (up to 12,000 specimens) a few mosquitoes (up to 4 specimens) were found positive for *Plasmodium* parasites even in their high abundance months [11, 12]. So, the question remains on how much is people being exposed to mosquito bites and acquiring the parasite. Thus, it is important to design alternative methods able to reflect the vector-human contact and complement the data collected by mosquito trapping methods.

Malaria is acquired when *Plasmodium* spp. sporozoites are injected into human skin through the bite of a female *Anopheles* along with the mosquito salivary proteins [22]. Previous studies have shown that a significant number of mosquito salivary proteins are immunogenic and able to induce antibody responses, mainly IgG isotype. These antibodies can reflect the intensity of human exposure to mosquito bites and represent good indicators of the risk of infection with *Plasmodium* spp. [23–27]. Thus, the use of salivary gland and saliva antigens has been previously validated as an indirect proxy to determine mosquito bite exposure. Significant higher IgG antibody levels against *An. albimanus* and *An. darlingi* salivary proteins have been observed in people with active malaria infection in Central and South America when compared to uninfected people living in the same region [23, 28]. A similar pattern has been observed in areas where *Anopheles* (*Cel.*) *gambiae* and *Anopheles* (*Cel.*) *stephensi* are among the most important vectors. A significant number of these studies were performed evaluating IgG responses against the *An. gambiae* salivary protein gSG6, a highly conserved protein among *Anopheles* species from the Subgenus *Cellia* and *Anopheles* [29]. The peptide, gSG6-P1, was designed from the original *An. gambiae* gSG6 sequence. IgG responses specific to this salivary peptide has been validated as a biomarker of human exposure not only in Africa but also in Asia and South America [24, 27, 30]. Although there are no known species of the subgenus *Cellia* in South America, the responses observed against the gSG6-P1 peptide could be hypothesized to result from the presence of mosquitoes belonging to the subgenus *Anopheles* such as *Anopheles pseudopunctipennis* and *An. punctimacula* and *An. calderoni* [31].

Consequently, it is necessary to characterize a broader panel of biomarkers able to identify the risk of disease more closely in areas with a great diversity of *Anopheles* mosquitoes. Future studies are planned to identify exposure markers that include not only the primary malaria vectors but also markers for the majority of the circulating species playing an important role in malaria transmission in Latin America, even when these vectors species are in a smaller proportion. Since the use of salivary gland extract as antigen to indirectly measure exposure to mosquito species circulating in a region has been validated by several groups the main objective of this work was to measure IgG antibodies in humans living in an area where low-density *P. falciparum* infections are frequent. Thus, human IgG responses to *Anopheles* salivary gland extracts (SGE) were used to measure potential associations with low-density infections by *P. falciparum* and malaria risk. Additionally, it was evaluated whether gSG6-P1 peptide continues as a useful marker to detect exposure in areas where mosquitoes from the sub-genus *Anopheles* are important vectors of malaria in Colombia.

## Methods

### Samples selection

The samples used in this study were collected as part of a longitudinal study in which the purpose was to evaluate the dynamic of submicroscopic *Plasmodium* infections in Colombia.

**Fig. 1:**
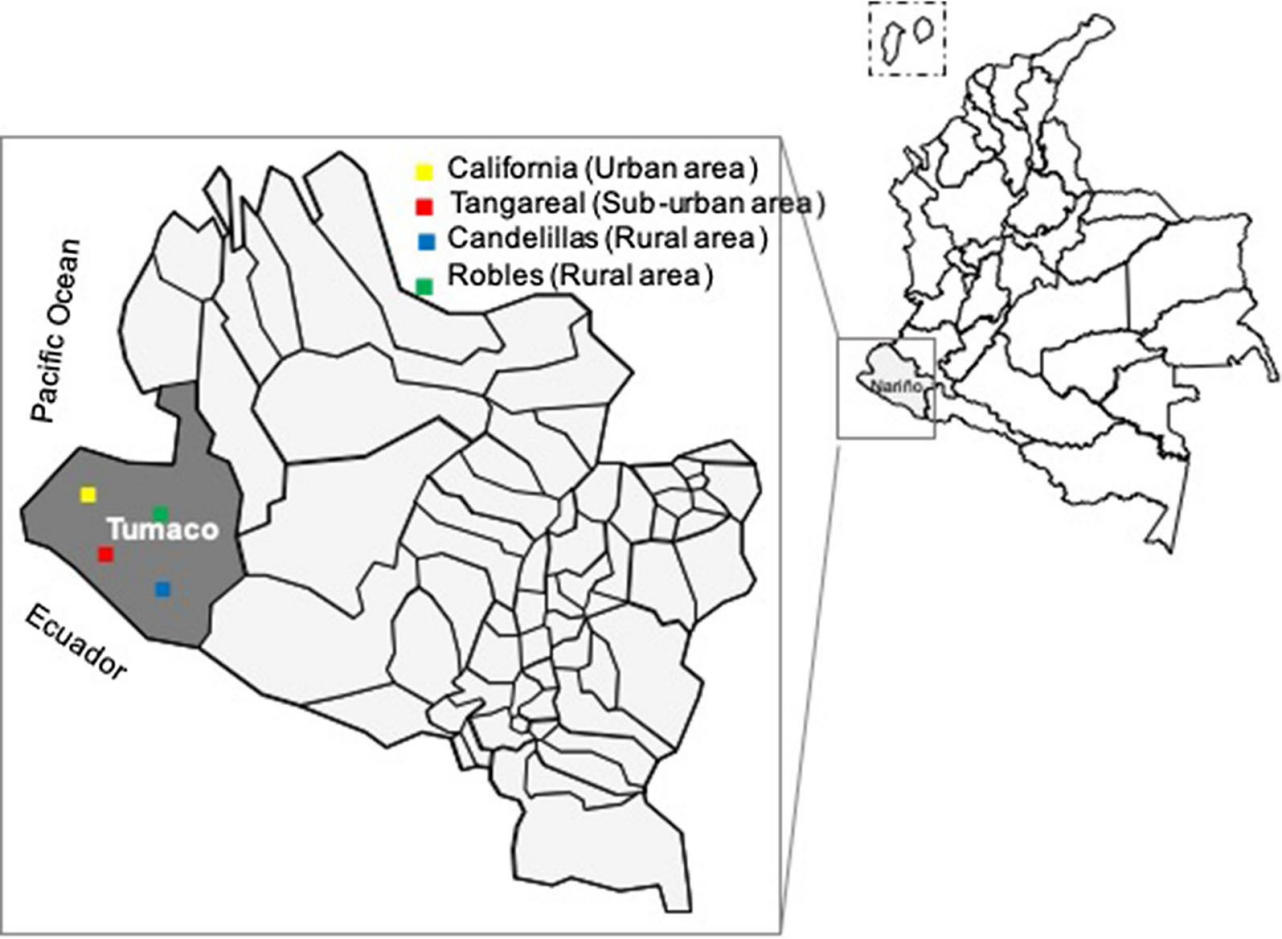
Study sites in Tumaco, Nariño, Colombia

Dried blood spots (DBS) in Whatman^®^ 903 protein saver card (GE Healthcare, US) were collected by passive case detection in the transversal phase of the study, conducted between August 2017 to March 2018 in four villages (California, Tangareal, Robles, and Candelillas) in Tumaco city located in the south of Colombia (1850′N, 78845′W) (Fig. 1). The first village represents a typical suburban zone. The following two sites are characterized as rural areas, and the last one is classified as a peri-urban zone. During the study, *P. falciparum* was reported as the predominant species (96%) in Tumaco with an API of 13.5 cases/1000 inhabitants in 2017 and 10.4 cases/1000 inhabitants in 2018. No entomological data was collected during the time of this study [32].

To compare the vector exposure between infected and uninfected individuals, all positive *P. falciparum* samples were selected (n = 63) from the 958 people that were enrolled in the main study. All of these infections were afebrile (axillary temperature < 37.5 °C), and 48 (76.2%) were submicroscopic (detected by Loop-mediated isothermal amplification-LAMP or nested polymerase chain reaction- nPCR but not by light microscopy-LM). Furthermore, 50 uninfected samples were randomly selected by age (± 5 years) and gender from the total of non-infected individuals by using an Excel random list.

### ELISA antigens and SGE preparations

*Anopheles albimanus* and *An. darlingi* were maintained under insectary conditions until salivary gland dissection. Based on recent studies suggesting that time of colonization has an influence on arthropod salivary gland content [33], and that two different *An. albimanus* lineages are circulating in two geographically distant regions of Colombia, the potential differences in antibody responses against salivary content of two different strains of *An. albimanus* were evaluated, one from a long-stablished colony strain STECLA (STE) versus a recently colonized strain Cartagena (CTG). Briefly, *An. albimanus* strains originated from El Salvador (STE) and Colombia (CTG), respectively, and were maintained in the insectary at the CDC (Atlanta, GA, USA). The *An. darlingi* laboratory strain originated from Iquitos, Peru [34], and was maintained in the NAMRU-6 insectary (Iquitos, Loreto, Peru). Salivary glands from 8 to 10 days old female mosquitoes were extracted by dissection and pooled into 1× PBS [23]. Mosquitoes were blood feed at day 3 or 4 after emergence. A pool of 100 salivary gland pairs from each strain was then frozen and thawed three times to prepare the SGE. The concentration of the SGE was determined using a NanoDrop™ (Thermo Scientific, Wilmington, DE, USA) and 50 µL aliquots were stored at − 80 °C until use. The *An. gambiae* gSG6-P1 peptide was synthesized by Genscript (Piscataway, NJ, USA) and the *P. falciparum* Pf-MSP (*Plasmodium falciparum* Merozoite Surface Protein) peptide (Fitzgerald, USA) was used to evaluate exposure to malaria parasites.

### Indirect ELISA (enzyme linked immunosorbent assay)

ELISA conditions were standardized as described elsewhere [23, 24]. Also, DBS samples were prepared as by eluting half of a card circle into 300 µL of elution buffer (PBS 1×, Tween 20 0.05%) and incubated overnight at 4 °C. Testing of serial dilutions (1:50, 1:100 and 1:200) showed better performance of the ELISA using a 1:50 dilution. Briefly, Nunc-Maxisorp 96-well plates (Nalgene Nunc International, Rochester, NY) were coated with 50 µL/well of gSG6-P1 peptide (2 μg/mL), *An. darlingi* and *An. albimanus* SGE (1 μg/mL) or Pf-MSP (1 μg/mL) diluted 1× PBS. Plates were incubated overnight at 4 °C and blocked with 200 µL of 5% skim milk solution in PBS-tween 20 (0.05%) (Blocking buffer) for 1.5 h at 37 °C. The DBS elution was used to prepare a 1:50 sample dilution in blocking buffer, this optimal dilution had been determined by preliminary experiments and 50 µL of diluted samples were added to each well (individual samples were tested in duplicate). Plates were incubated at 37 °C for 1.5 h, washed three times, then incubated 1 h at 37 °C with 50 µL/well of a 1/1000 dilution of goat monoclonal anti-human IgG conjugated with horseradish peroxidase (AbCam, Cambridge, MA). After three final washes, colorimetric development was carried out using tetra-methyl-benzidine (Abcam) as a substrate. In parallel, each assessed microplate contained in duplicate: a positive control, a negative control, and a blank; wells containing no sample. The positive control was a pool of DBS of people with positive malaria diagnosis. The negative control was a sample of people from US (n = 36) with no exposure to malaria parasites. The blank was composed by wells containing no sample. The reaction was stopped with 0.25 N sulfuric acid, and the optical density (OD) was measured at 450 nm.

### Statistical analysis

All data from questionnaires and forms were entered into a Microsoft Access database, and statistical analyses were conducted in STATA 14 (StataCorp. 2015. Stata Statistical Software: Release 14. College Station, TX: StataCorp LP) and GraphPad Software V5. OD normalization and plate to plate variation was performed as described elsewhere [24]. Briefly, antibody levels were expressed as the ΔOD value: ΔOD = ODx − ODb, where ODx represents the mean of individual OD in both antigen wells and ODb the mean of the blank wells. For each tested peptide, positive controls of each plate were averaged and divided by the average of the ODx of the positive control for each plate to obtain a normalization factor for each plate as previously described. Each plate normalization factor was multiplied by plate sample ΔOD to obtain normalized ΔOD that were used in statistical analyses. Assay variation of samples (inter and intra assay) tested in the study was below 20% and it was only included in the analysis serum samples with a coefficient of variation ≤ 20% duplicates between duplicate [35]. The mean ΔOD of negative US controls plus 3 standard deviations (SD) was used to determine cut-off value for responsiveness to antigens. The ΔOD cut off value to determine exposure to malaria antigens as 0.263. The median of antibody level for each antigen in uninfected people (negative PCR and negative LM) in submicroscopic (positive PCR and negative LM) and microscopic (positive PCR and positive LM) carriers was estimated. The medians are shown with their respective interquartile ranges (IQR).

Odd ratios (OR) were calculated to evaluate risk of malaria. For this, the median was used to classify IgG antibody levels as high (ΔOD higher than the median) and low (ΔOD equal or lower than the median) and the samples were classified as cases (Asymptomatic and submicroscopic infections) and controls (uninfected). In addition, Spearman correlation coefficients were calculated to measure the strength of association between each *Anopheles* antigen with Pf-MSP IgG levels. Finally, a Mann–Whitney U-test was used to estimate differences between medians of each *Anopheles* antigen by the status of infection in the whole sample and by sites and a Kruskal–Wallis test to estimate differences between groups of infection. A multiple linear mixed regression model was constructed to determine the correlation between anti-*Anopheles* IgG levels (anti-gSG6-P1, CTG, STE, and *An. darlingi*) with anti Pf-MSP IgG levels. A random intercept at the village level was introduced in the model to correct the inter-village variations. The model was adjusted by *Plasmodium* infection, age and time of residence in a malarial endemic area; these factors showed significant p values in simple models.

## Results

### Study sample demographics, sociocultural variables and antibody responses to mosquito antigens

The exposure to mosquito bites in the area of Tumaco in Nariño (Colombia) (Fig. 1) was studied shows the characteristics of participants according to the status of infections. The gender and age groups distribution seem to be equally represented between infected and uninfected individuals. The majority of infected people came from California and Tangareal (78.7%). There was a higher proportion of people with malaria history on infected people (42/63, 66.6%) compared uninfected people group (25/50, 50%), and 33.0% of them, got at least one episode of malaria in the previous year. Pairwise comparison of the level of IgG antibodies against *An. albimanus* (STE and CTG), *An.* (*Nys.*) *darlingi* or gSG6-P1 by gender, education level and occupation did not show significant differences (Mann–Whitney test p > 0.05).

### Detection of IgG antibody against *Anopheles* SGE and gSG6-P1 peptide by infection status

**Fig. 2:**
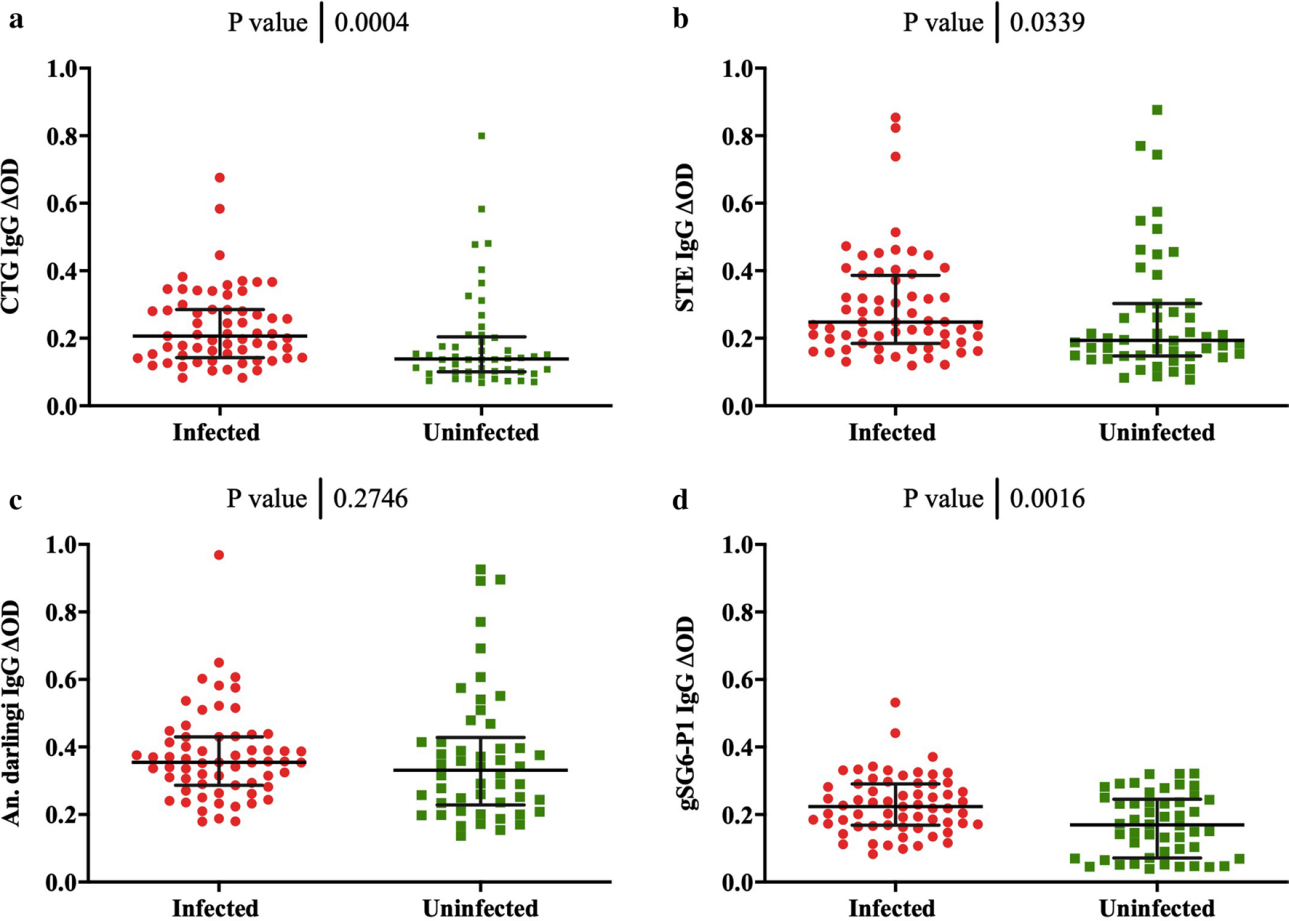
IgG responses to *Anopheles* per status of infection. **a** The individual anti CTG IgG levels, **b** STE, **c** *An.* (*Nys.*) *darlingi* SGE and** d** gSG6‑P1 peptide. Legend: Horizontal lines in the boxes indicate median values; lengths of boxes correspond to the inter-quartile ranges. Pairwise significance was tested with Mann–Whitney test

The level of antibodies against *An. albimanus* salivary proteins from both strains (STE and CTG) and against the gSG6-P1 peptide was significantly higher in volunteers with *Plasmodium* infection (CTG, Mann–Whitney test *p *=* 0.0004*; STE, Mann–Whitney test *p *=* 0.033*; and gSG6-P1, Mann–Whitney test *p *= 0.0016) antibody levels (Fig. 2). However, this difference was not observed when testing IgG antibodies against the whole SGE from *An. darlingi* (Mann–Whitney test p value = 0.2746). This is consistent with information provided by previous studies showing *An. albimanus* as one of the important vectors in the region.

**Fig. 3:**
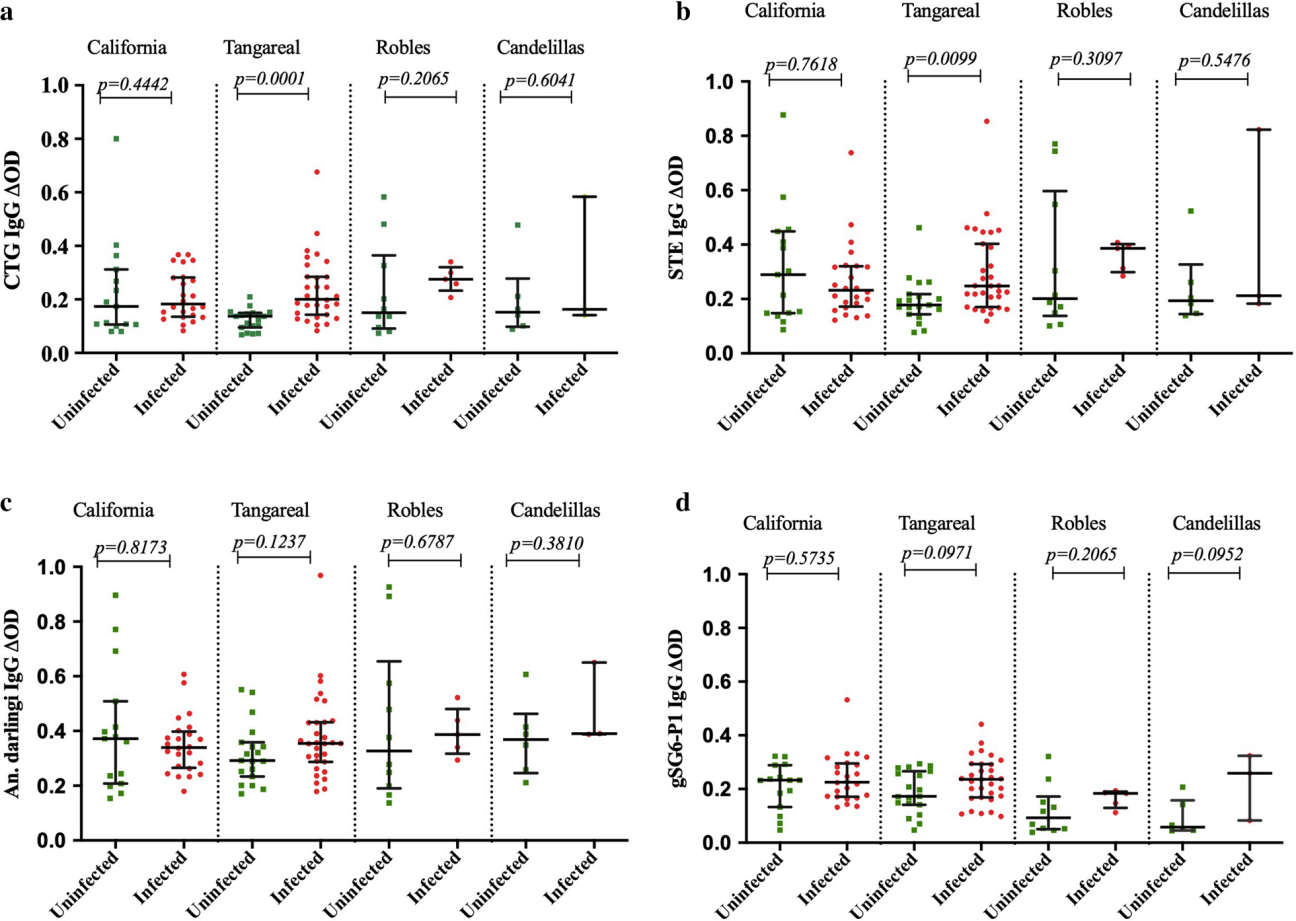
IgG responses to *Anopheles* per status of infection and per site. **a** The individual anti CTG IgG levels, **b** STE, **c** *An.* (*Nys.*) *darlingi* and **d** gSG6‑P1 peptide. Horizontal lines in the boxes indicate median values; lengths of boxes correspond to the inter-quartile ranges. Pairwise significance was tested with Mann–Whitney test

Figure 3 shows the difference observed in antibody level between infected and uninfected by the village where samples were collected. Except for the California neighborhood, the IgG levels in infected samples were higher than uninfected. Nevertheless, there were only significant associations for CTG and STE in Tangareal village. When the risk of suffering a malaria infection was calculated, it revealed a significantly higher risk of suffering malaria if the patient present higher levels of antibodies against CTG (OR = 3.4, 95% CI 1.468–8.131, Fisher’s Exact test *p *= *0.0023*), STE (OR = 2.68, 95% CI 1.166–6.234, Fisher’s Exact test *p *= *0.138*) and gSG6 = P1 (OR = 2.30, 95% CI 1.009–5.309, Fisher’s Exact test *p *= *0.0374*) but not for *An. darlingi* SGE (OR = 1.4, 95% CI 0.656–3.349, Fisher’s Exact test *p *= *0.3454*).

### Detection of IgG antibody levels by *P. falciparum* detection threshold (microscopic *vs.* sub-microscopic)

**Fig. 4:**
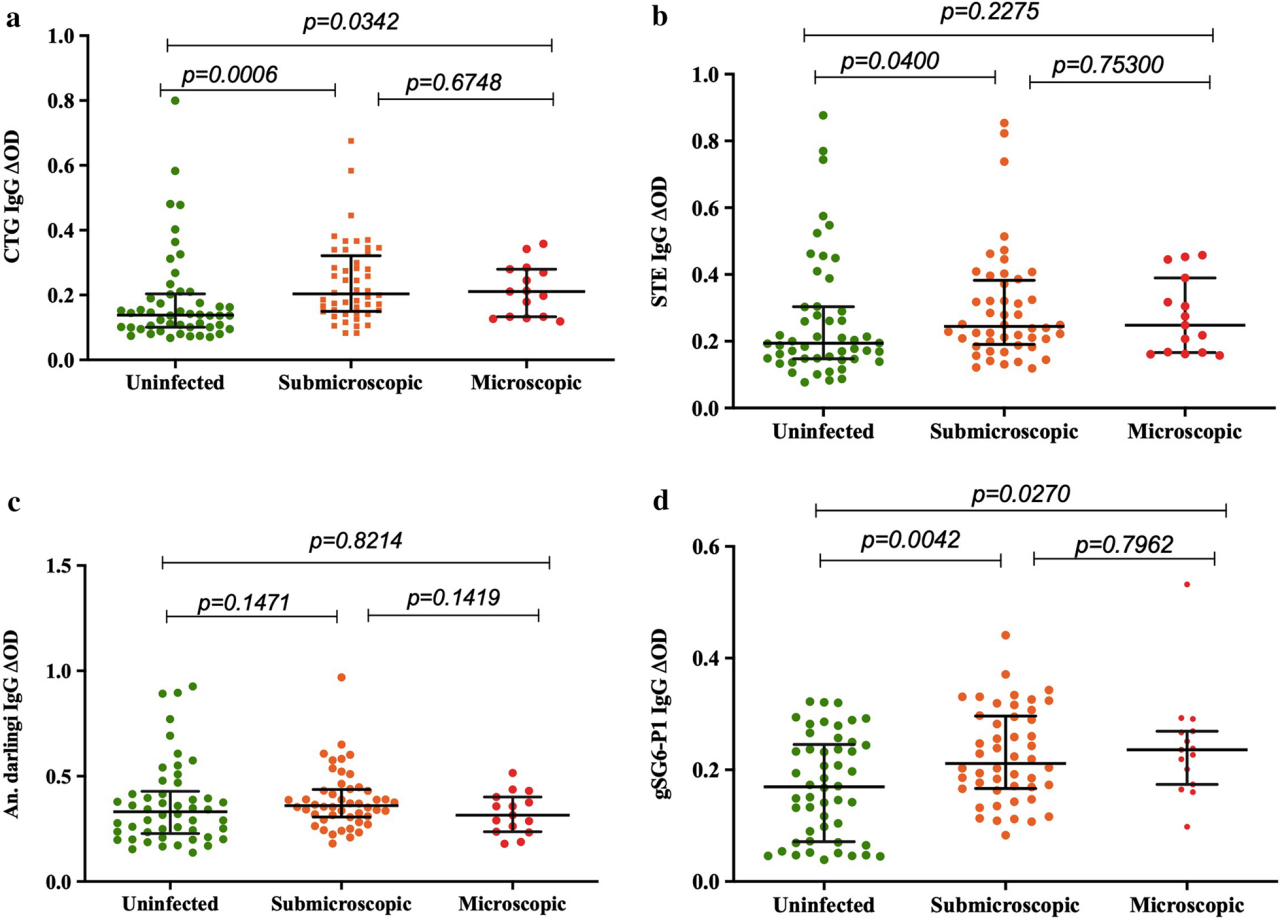
IgG responses to *Anophele*s per infection group: uninfected, submicroscopic (positive PCR and negative LM) and microscopic (positive by both PCR and LM). **a** The individual anti CTG IgG levels, **b** STE, **c** *An.* (*Nys.*) *darlingi* and **d** gSG6‑P1 peptide. Horizontal lines in the boxes indicate median values; lengths of boxes correspond to the inter‑quartile ranges. Pairwise significance was tested with Mann–Whitney test

All of *Plasmodium* infected patients were afebrile and considered as asymptomatic carriers. However, they were grouped according to the diagnostic test results into microscopic (if parasites were detected by LM and PCR) or submicroscopic if parasites were only detected by PCR (Fig. 4). Accordingly, results showed that IgG levels might change according to parasitaemia. Specifically, it was observed a tendency of increased antibody levels in samples where parasitaemia was detected by light microscopy compared to infections only detected by molecular tests and also in uninfected specimens. There were significant differences in the median IgG antibody levels against CTG (Kruskal–Wallis test *p *= *0.0016*) and gSGS-P1 (Kruskal–Wallis test *p *= *0.0067*) between the three groups of infections. Although the tendency was also observed when using STE and *An. darlingi* as antigen, the differences were not significant (Table 1).

**Table 1:** Socio-demographic characteristics, malaria history and IgG levels in the study population

Characteristic	Uninfected		Asymptomatic malaria		Total	
	N = 50		n = 63		n = 113	
	n	%	n	%	n	%
Age						
< 5	4	8.0	3	4.8	7	6.2
5–15	16	32.0	17	27.0	33	29.2
> 15	30	60.0	43	68.3	73	64.6
Site						
California	15	30.0	24	38.1	39	34.5
Tangareal	19	38.0	31	49.2	50	44.2
Robles	10	20.0	5	7.9	15	13.3
Candelillas	6	12.0	3	4.8	9	8.0
Gender						
Male	20	40.0	28	44.4	48	42.5
Female	30	60.0	35	55.6	65	57.5
Episodes of malaria						
0	25	50.0	21	33.3	46	40.7
1	11	22.0	16	25.4	27	23.9
> 1	14	28.0	26	41.3	40	35.4
Malaria last year						
No	38	76.0	42	66.7	80	70.8
Yes	12	24.0	21	33.3	33	29.2
Education level						
High school or lower	35	70.0	49	77.8	84	74.3
Undergraduate or graduate	15	30.0	14	22.2	29	25.7
Occupation						
Housewife	14	28.0	19	30.2	33	29.2
Farmer	4	8.0	9	14.3	13	11.5
Student	19	38.0	24	38.1	43	38.1
Others	13	26.0	11	17.5	24	21.2

IgG levels (ΔOD^a^)	Median (IQR^b^)	Median (IQR)	Median (IQR)
*An.* (*Nys.*) *darlingi*	0.332 (0.234–0.415)	0.355 (0.287–0.430)	0.352 (0.258–0.430)
CTG	0.139 (0.101–0.202)	0.207 (0.143–0.285)	0.172 (0.126–0.275)
STE	0.194 (0.148–0.303)	0.248 (0.185–0.386)	0.219 (0.168–0.324)
gSG6-P1	0.170 (0.072–0.244)	0.224 (0.169–0.291)	0.203 (0.141–0.267)

^a^Normalized optical density

^b^Interquartile range

### Association between exposure to *Anopheles* antigens and antibodies against *Plasmodium* Pf-MSP1 protein

**Fig. 5:**
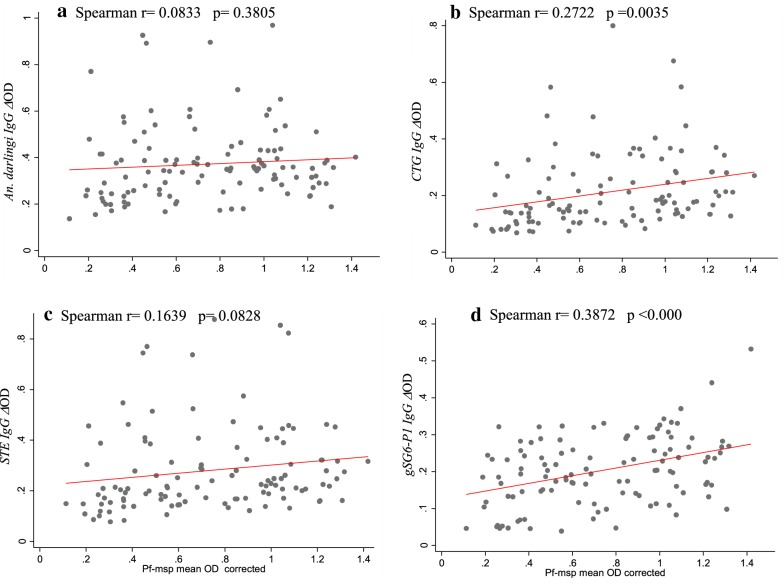
Correlation between anti- *Anopheles* IgG levels and anti-*Plasmodium* IgG levels in the whole population. Legend: Anti CTG and anti-Pf-MSP (**a**), STE and anti-Pf-MSP (**b**), *An.* (*Nys.*) *darlingi* and anti-Pf-MSP (**c**), and gSG6-P1 anti-Pf-MSP (**d**). The red solid line indicates the correlation curve

When evaluating whether there was any correlation between the level of IgG antibodies against the Pf-MSP1 protein and exposure to mosquito bite reflected by the levels of IgG antibodies against the salivary antigens, it was observed a positive association between Pf-MSP IgG levels with anti CTG (Spearman r = 0.2722, *p *= *0.0035*) and gSG6-P1 peptide (Spearman r = 0.3872; *p *< *0.001*) (Fig. 5), but not for *An. darlingi* and STE SGE.

### Antibody-based model to evaluate factors of variation in responses against *Anopheles* and *Plasmodium* antigens

Independent of location (random intercept at village levels), IgG response to CTG and gSG6-P1 peptide were positively associated with the IgG response to *P. falciparum* (regression coefficient (RE) = 0.105; 95% CI 0.0223–0.189 and RE = 0.070; 95% CI 0.013–0.126, respectively). In contrast with the IgG Pf-MSP, a negative association for all IgG responses to *Anopheles* was found with age showing there is a decreasing of IgG immune response with increased age (Table 2 Linear Mixed Effects models to explain exposure to *Anopheles* in a malaria-endemic area in Colombia). A similar situation occurred with the time of residence in an endemic area for malaria; IgG responses to gSG6-P1 peptide was 3.4% lower in samples from people who had lived in a malarial area for more than 5 years (RE = − 0.035; 95% CI − 0.070 to − 0.003). Finally, no significant variation of specific anti-*Anopheles* IgG was observed according to the status of infection (Table 2 Linear Mixed Effects models to explain exposure to *Anopheles* in a malaria-endemic area in Colombia).

**Table 2:** Linear mixed effects models to explain exposure to *Anopheles* in a malaria-endemic area in Colombia

	Anti-*An. darlingi* IgG			Anti-CTG IgG			Anti-STE IgG			Anti-gSG6-P1 IgG		
	Estimated coefficient	SE	95% CI	Estimated coefficient	SE	95% CI	Estimated coefficient	SE	95% CI	Estimated coefficient	SE	95% CI
Fixed effects												
Intercept	0.428	0.046		0.191	0.036		0.313	0.047		0.128	0.024	
Infectious status												
Uninfected	1			1			1			1		
Infected	− 0.022	0.037	− 0.096 to 0.051	0.007	0.029	− 0.051 to 0.064	− 0.005	0.038	− 0.080 to 0.069	0.021	0.020	− 0.018 to 0.060
Age	− 0.003	0.001	− 0.004 to − 0.001	− 0.002	0.001	− 0.003 to − 0.001	− 0.003	0.001	− 0.005 to − 0.001	0.001	0.000	0.000 to 0.002
Residence time												
< 5 years	1			1			1			1		
≥ 5 years	− 0.031	0.030	− 0.090 to 0.029	− 0.007	0.024	− 0.054 to 0.040	− 0.032	0.031	− 0.093 to 0.029	− 0.035	0.016	− 0.070 to − 0.003
Pf-MSP	0.066	0.054	− 0.041 to 0.172	0.106	0.043	0.022 to 0.189	0.092	0.055	− 0.017 to 0.200	0.070	0.029	0.013 to 0.126
Village level	6.51E−22	1.56E−20		4.31E−27	9.71E−26		7.13E−27	1.48E−25		2.46E−05	1.96E−04	

## Discussion

The intensity of malaria transmission has been traditionally evaluated using the EIR, which is defined by the number of infected bites received per human per unit of time; nevertheless, this strategy has shown limitations in low endemic settings for malaria [25, 36]. As a result, alternative methods to estimate human exposure to *Anopheles* bites have been proposed, including the detection of IgG responses to *Anopheles* SGE and salivary peptides. The purpose of the present study was to explore the possibility of using whole SGE from different *Anopheles* species as tool to detect IgG antibodies in humans that could be used as indirect estimation of exposure to *Anopheles* bites in a malaria-endemic area in Colombia where there is an important proportion of asymptomatic infections. Based in previous reports suggesting at least two *An. albimanus* lineages in Colombia [7, 9, 10], the SGE from two *An. albimanus* strains were used to try to capture potential differences in immunogenicity of salivary proteins from colony mosquitoes isolated from different geographical regions and with differences in the colonization time. Specifically, this study includes the comparison of salivary gland content immunogenicity between the CTG strain, a recently colonized strain, that could potentially resemble more closely responses to “wild mosquito antigens” in the area, to the immunogenicity displayed by the STE strain, isolated in Central America in 1974.

*Anopheles albimanus* has been reported as one of the main malaria vectors in Nariño displaying EIR up to 2.6 in recent studies. Consistent with previous studies, *An. albimanus* SGEs (STE and CTG) were associated with the infectious status, where people with active *Plasmodium* infection presented significantly higher IgG antibody levels against the salivary proteins. This study also showed that people with higher antibody levels against STE, CTG and gSG6-p1 have between 2 and 4 times more probability of suffering a malaria infection. These results agree with previous findings in Haiti were the IgG antibody levels against *An. albimanus* SGE were higher in patients with clinical malaria than those in uninfected people living in the same region [23]. These studies suggest that the IgG antibody response against *An. albimanus* SGE is associated with *Plasmodium* exposure and highlights the relevance of using whole salivary content in the form of SGE as potentially useful antigen to measure risk of infection in areas of low and seasonal transmission. Interestingly, the relationship between parasitaemia and IgG antibodies against *Anopheles* antigens was significant when using the antigen from the CTG strain and not the STE, suggesting that the antigens contained on the SGE from the CTG may be more closely related to the one the study subjects are exposed in the field. However, no association was found between antibodies levels against *An. darlingi* SGE and malaria infection. This could be explained due to the low abundance (or probable absence) of *An. darlingi* mosquito previously reported in areas where samples were collected [6, 7]. Still, the observed antibody response against the *An. darlingi* SGE may be explained by a potential cross reactivity between salivary proteins present in mosquitoes from the subgenus *Nyssorhynchus*, which *An. darlingi* belongs to.

Previous studies suggest that *An. calderoni* is a primary malaria vector in Narino [11]. This may explain the current findings showing a high IgG response against gSG6-P1 peptide in samples from infected compared to uninfected people. These findings agree with a previous study in Colombian volunteers suggesting that the concentration of gSG6-P1 antibodies is significantly correlated with malaria infection status and that people with clinical malaria presented significantly higher levels of IgG anti-gSG6-P1 antibodies than healthy controls [24]. Although, *Anopheles* species from the subgenus *Nyssorhynchus* are the main vectors of malaria in Colombia, at least six species from the sub-genus *Anopheles* have been described as potential malaria vectors in the region [37, 38]. Three of these species (*An. calderoni*, *An. pseudopunctipennis* and *An. punctimacula*) are present along the Pacific coast, the main area where *P. falciparum* is transmitted in Colombia [3]. Although Arcà et al. reported that gSG6 had no degree of identity with orthologous proteins from vectors in Central and South America, and therefore serological data previously published about the usefulness of the gSG6-P1 peptide in Colombia [24] should be interpreted with caution [29], previous work also showed that a deduced gSG6 from the New World species *An. freeborni* and *An. quadrimaculatus* (from the subgenus *Anopheles*) had between 67 and 71% of degree of identity with the gSG6 from Old World *Anopheles* species [39]. In the same way, Pollard et al. suggested that the antibodies to the gSG6-P1 peptide in the Colombian population may represent exposure to *An. punctimacula*, which is a member of the *Anopheles* subgenus or could hypothetically represent exposure to minor vectors in the country [31]. Thus, the current results suggest that the gSG6-P1 peptide could be a useful marker for malaria risk in areas of Colombia where mosquitoes belonging to subgenus other than *Nyssorhynchus* are present.

When comparing IgG levels against *An. albimanus* among villages, it was observed that SGE from both STE and CTG, were higher in infected than uninfected people in all villages except California. This is interesting because California is an area with urban characteristics, unlike Tangareal which is a sub-urban area and Robles and Candelillas which are rural areas. To evaluate further, the multilevel analysis demonstrated that independent of site, both age and, anti-Pf-MSP IgG levels were associated not only with IgG antibody levels against the CTG strains of *An. albimanus* but also against the gSG6-P1. Suggesting the importance of using a panel of exposure biomarkers (mosquito antigens) and concurrent entomological data to accurately evaluate risk especially in areas where several *Anopheles* species are implicated in malaria transmission. Also, the current model described in this study revealed a negative association between age and IgG antibodies against all *Anopheles* antigens. Similar trend has been observed in other studies measuring antibody responses against mosquito salivary antigens and has been associated with the development of tolerance against certain mosquito allergens [35, 40, 41].

Recent studies revealed important differences in salivary content in arthropods collected in the field when compared to the same species maintained in a colony [33]. Also, a previous study suggests the possibility of two *An. albimanus* lineages circulating two geographically distant regions of Colombia. Thus, the aim of this study was to determine if the risk of infection can be affected by the salivary content of mosquitoes from the same species but from different origins. So, a recently colonized strain (CTG) and a long-term established laboratory colony (STE) each isolated from a distinct geographical region (Colombia and El Salvador) to account for potential changes in IgG responses based on salivary content were used. As the results indicate, the SGE from the CTG strain showed significant association with the Pf-MSP1 and not with the SGE from STE suggesting potential differences. Determination and confirmation of these differences are subject of further studies aimed to characterize salivary gland content of the two *An. albimanus* lineages circulating in Colombia and comparing those to *An. albimanus* isolates from other countries. This is important since the use of salivary antigens as vaccines for malaria are undergoing [42] and characterization of the main immunogenic salivary proteins of the main vectors circulating in endemic areas are important for the success of such vaccine.

This study has several limitations. First, because this study was cross-sectional, association with the anti-*Anopheles* IgG levels should be interpreted with caution as they do not imply causality. Second, due to the lack of a symptomatic group, it was not possible to determine the risk factors for this kind of infection and to explore the differences in the anti-*Anopheles* IgG levels between uninfected, asymptomatic (both, submicroscopic and microscopic infections) and symptomatic groups. Also, the lack of concurrent entomological data is a significant limitation. Since this study did not included mosquito collection or other concurrent entomological surveillance, the current results should be interpreted as an indirect measurement of disease risk (currently calculated by OR) until further determination of the specific mosquitoes circulating in an area where these antibodies are measured. A future study phase will include to complete the serological data with entomological data to further validate the findings of this study. Despite these limitations, these results are useful to identify new potential biomarkers for malaria risk in Colombia.

## Conclusion

This study demonstrates that SGE from *An. albimanus* strains CTG and STE could be a potential source of new *Anopheles* salivary biomarkers to determine risk of malaria in Colombia, supports previous findings that gSG6-P1 peptide has the potential to quantify human exposure to some malaria secondary vectors. All of them could be useful to estimate the risk of malaria transmission and could provide relevant tools to better understand malaria transmission dynamics and orient control strategies according to the specific characteristics in low-endemic settings.

## Data Availability

All data generated or analyzed during this study are included in this published article and its supplementary information files. The datasets used and/or analysed during the current study are available from the corresponding author on reasonable request.

## Abbreviations

IgG: immunoglobulin G
STE: STECLA
CTG: cartagena
EIR: annual entomological inoculation rate
API: Annual Parasite Index
VC: vectorial capacity
HLC: Human Landing Collection
CDC: Center for Disease Control
SGE: salivary gland extracts
DBS: dried blood spots
LAMP: loop-mediated isothermal amplification
nPCR: nested polymerase chain reaction
LM: light microscopy
Pf-MSP: *Plasmodium falciparum* Merozoite Surface Protein
ELISA: enzyme linked immunosorbent assay
IQR: interquartile range
OD: optical density
SD: standard deviations
IQR: interquartile range
OR: odds ratio
